# Exosomes miR-22-3p Derived from Mesenchymal Stem Cells Suppress Colorectal Cancer Cell Proliferation and Invasion by Regulating RAP2B and PI3K/AKT Pathway

**DOI:** 10.1155/2021/3874478

**Published:** 2021-06-21

**Authors:** Yan Wang, Changkun Lin

**Affiliations:** ^1^Departmeng of Gastroenterology, Shengjing Hospital of China Medical University, Shenyang 110004, China; ^2^College of Basic Medical Science, China Medical University, Shenyang 110122, China

## Abstract

**Objective:**

Exosomes (exo) which contain proteins, microRNAs (miRNAs), and other bioactive substances can participate in intercellular signal transduction and material transport. Bone marrow mesenchymal stem cells (BMSCs) have a strong ability to produce exosomes. The purpose of this study was to observe the effect of hBMSCs-derived-exo miR-22-3p on proliferation and invasion of colorectal cancer (CRC) cells and to explore its mechanism.

**Methods:**

miR-22-3p and RAS oncogene family (RAP2B) expression was detected using qRT-PCR or Western blotting. Their interaction was confirmed by dual luciferase activity assay. Effects of miR-22-3p on cell proliferation and invasion were evaluated by CCK-8 and Transwell assay, respectively. Exosomes were extracted by the ultracentrifugation and identified through electron microscopy and Western blotting.

**Results:**

In CRC tissues and cells, downregulation of miR-22-3p and upregulation of RAP2B were observed. According to the analysis of dual luciferase activity, RAP2B was a target gene of miR-22-3p. In addition, miR-22-3p obviously repressed the cells proliferation and invasion via mediating RAP2B/PI3K/AKT pathway. Coculture experiments indicated that miR-22-3p derived from hBMSCs-exo had inhibition effects on SW480 cell proliferation and invasion.

**Conclusions:**

Collectively, miR-22-3p from hBMSCs-exo might impede CRC progression, which emphasized the potential of hBMSCs-exo-miR-22-3p as CRC treatment in the future.

## 1. Introduction

Colorectal cancer (CRC) is one of the common malignant tumors of the digestive tract with higher global morbidity and mortality [1]. With the changes in the diet structure of the Chinese people, the incidence of CRC has been increasing, and it has become a major tumor that threatens the health of our population [2]. Because of the relatively insidious onset and the atypical early symptoms of CRC, most patients are in the advanced stage of clinical diagnosis and miss the best opportunity for treatment [3]. Although the existing diagnosis and treatment methods have continued to be improved, the overall prognosis and the 5-year survival rate of the patient are still poor [4]. CRC is a complex process involving many factors and many steps, and an in-depth discussion of its pathogenesis has great significance for the early diagnosis and treatment of CRC patients [5].

Exosomes are vesicles with a size of 30–200 nm derived from the cell membrane of normal or cancerous cells and released into various body fluids, such as functional extracellular fluids (blood, lymph fluid, tissue fluid, etc.), urine, breast milk, saliva, and bile [6]. These vesicles can be transferred from the donor to the recipient cells and play a key role in intercellular communication [7]. The contents of exosomes have different components, such as DNA, RNA, lipids, and proteins, which can reflect the metabolism, cancerization, and apoptosis of cells to some extent [8]. Among them, miRNAs are the most studied exosomes-mediated small biomolecules. As a type of single-stranded noncoding RNA with a length of 18 to 25 nucleotides, miRNAs participate in different processes of malignant tumors, such as cancer cells proliferation, angiogenesis, and tumor metastasis [9]. Additionally, lots of researches reported that miRNAs regulated the progression of CRC and cell proliferation, invasion, and metastasis by regulating specific target genes expression [10]. miR-22-3p, as a tumor suppressor, has been reported to repress HCC cell proliferation through downregulating the target gene Spl [11]. Moreover, miR-22-3p may inhibit NSCLC cell proliferation via targeting AEG-1 [12]. However, there are few studies on the role of miR-22-3p in CRC.

Mesenchymal stem cells (MSCs) with immunosuppressive, immunomodulatory, and therapeutic properties are the cells with the strongest ability to secrete exosomes, and MSC-exosomes have many biological functions similar to MSCs [13]. BMSCs, which have immunogenicity, high portability, and multidirectional differentiation ability, are an example of the most widely studied MSCs [14]. hBMSCs act as regulators of apoptosis, angiogenesis, and immune tolerance in tumors [15]. They have significant tumor localization and immune specificity and can be used as a carrier to deliver antitumor drugs to the tumor microenvironment [16]. In recent years, increasing researches were focused on the function of miRNAs derived from BMSCs exosome in various cancers progression, including CRC. For example, miR-16-5p derived from BMSCs-exosomes repressed CRC cell biological function via ITGA2 [17]. Meanwhile, Chen et al. found that BMSCs-derived exosome miR-4461 impeded CRC tumorigenesis through inhibiting the expression of COPB2 [18]. On the basis of previous researches, we supposed that hBMSCs-derived exosomes miR-22-3p might be concerned with CRC progression. Herein, we conducted this study to verify the above hypothesis and explore the potential mechanism.

## 2. Materials and Methods

### 2.1. Tissue Specimens

36 pairs of CRC tissues (identified by pathological diagnosis) and matched adjacent tissue samples were obtained from Shengjing Hospital of China Medical University from August 2018 to June 2019. All patients received no radiotherapy or chemotherapy before treatment and had no history of other malignant tumors. All tissue samples were immediately snap-frozen using liquid nitrogen and preserved in −80°C for experiments. All patients provided written informed consent and the study was approved by the ethics committee of Shengjing Hospital of China Medical University.

### 2.2. Cell Culture

NCM460 cells (human normal colon mucosal epithelial cell line), human CRC cell lines (HT-29, SW620, HCT-116, SW480, and LoVo), and hBMSCs were purchased from American Type Culture Collection (MD, USA). hBMSCs were cultured in DMEM/F12 (supplemented with 10% fetal bovine serum (FBS, Gibco, USA)) and 1% penicillin-streptomycin) medium (Gibco, USA). Exosomes were removed from FBS by ultracentrifugation. The resuspended hBMSCs were placed in a humid environment at 37°C and 5% CO_2_. When the adherent hBMSCs reached 80% confluence, the supernatant was collected and expanded to obtain hBMSCs. All the other cells were cultured in DMEM containing 10% FBS at 37°C in an incubator with 5% CO_2_.

### 2.3. Transfection

Plasmids for SW480 cells transfection containing miR-22-3p mimic and inhibitor and their control (miR-NC and anti-NC), RAP2B overexpression vector pcDNA3.1-RAP2B (pc-RAP2B), and pcDNA3.1 empty vector (pc-NC) were obtained from Shanghai GenePharma in China. hBMSCs at passages 2-3 were transfected with miR-NC, anti-NC, and miR-22-3p mimic and inhibitor, respectively. All transfections were conducted by Lipofectamine 2000 (Invitrogen, USA). The exosomes isolated from transfected hBMSCs (exo-miR-NC, exo-anti-NC, exo-mimic, and exo-inhibitor) were cocultured with SW480 cells for 48 h, respectively.

### 2.4. Isolation of hBMSCs Exosomes

Transfected hBMSCs were inoculated into a culture flask with 5 × 10^6^ cells/75 cm^2^, and 10 ml serum-free medium was added. After 3 days of culture, serum-free medium was collected and centrifuged at 10,000 r/min for 30 minutes to remove cell residues and impurities, and the supernatant was aspirated. Then we added exosome extraction reagent to supernatant and mixed thoroughly to incubate at 4°C overnight. The precipitate obtained by centrifugation is exosome. The precipitate was resuspended in PBS and stored at −20°C. Finally, we observed and photographed with a transmission electron microscope and detected its antigen expression by Western bolt.

### 2.5. Cell Proliferation Assays

Cells of each group were seeded in 96-well plates with 2 × 10^4^/well and cultured at 37°C with 5% CO_2_. At 24, 48, 72, and 96 h of culture, 10 *μ*l of CCK-8 solution (Sigma, USA) was added to each well and it was incubated for 2 h. Finally, a spectrophotometer was used to measure the absorbance at 450 nm.

### 2.6. Transwell Invasion Assay

500 *μ*l of RPMI 1640 culture medium containing 10% FBS was added to the lower chamber coated with Matrigel (Corning, USA), and 200 *μ*l of serum-free cell suspension (2.5 × 10^5^ cells/ml) was added to the upper chamber. Transwell chamber was cultured at 37°C, 5% CO_2_ for 24 h. Then, cells were fixed with 4% paraformaldehyde solution for 20 min, washed with PBS, and stained with 0.1% crystal violet. We observed the cells under Olympus inverted fluorescence microscope and counted the number of cells passing through Matrigel polymer gel and cell microporous membrane.

### 2.7. Dual Luciferase Activity Assay

Bioinformatics prediction software TargetScan is used to predict the target genes of miR-22-3p. RAP2B was selected as the research object. In order to further verify the targeting relationship between miR-22-3p and RAP2B, 3'-UTR luciferase reporter vectors of wild-type RAP2B (RAP2B-wt) and mutant RAP2B (RAP2B-mut) were constructed and cotransfected with miR-22-3p mimic, respectively. The dual luciferase detection kit (Promega, USA) was used to detect the dual luciferase activity.

### 2.8. Quantitative Real-Time PCR

Total RNA was extracted by TRIzol reagent (Invitrogen, USA). According to the reverse transcription kit (TaKaRa, Japan) instructions, reverse transcription reaction was performed to synthesize cDNA strands, and SYBR green (TaKaRa, Japan) was added for qRT-PCR amplification on ABI 7500 System (ABI, USA). U6 or GAPDH was used as internal reference, and the relative expression of RNAs was calculated by the 2^−ΔΔCt^ method. Primers are listed in Table 1.

### 2.9. Western Blot

RIPA lysate was used to extract the total protein from cells, and the protein concentration was determined by BCA method. 30 *μ*g protein was subjected to 10% SDS-PAGE. After protein separation, it was transferred to PVDF membranes. The transformed membrane was blocked with 5% skimmed milk. Then membranes were incubated with rabbit anti-CD9 (1 : 2000 dilution), rabbit anti-CD63(1 : 2000 dilution), rabbit anti-Hsp70 (1 : 2000 dilution), rabbit anti-RAP2B (1 : 1500), rabbit anti-PI3K (1 : 1500), rabbit anti-AKT (1 : 1500), rabbit anti-p-AKT (1 : 1500), and mouse anti-GAPDH (1 : 10,000 dilution) (Cambridge, USA) at 4°C overnight. After washing the membrane, incubation of HRP-labeled goat anti-rabbit or mouse secondary antibody (1 : 5000 dilution) was performed for 1 h at room temperature. Enhanced chemiluminescence (ECL) was used for development of the protein bands, and images were collected in the gel imaging system.

### 2.10. Statistical Analysis

GraphPad Prism v6.0 (GraphPad Software Inc, CA) software was used to do statistical analysis. Data in this study were expressed as mean ± standard deviation (SD). The *t*-test was used for comparison between two groups, and the one-way analysis of variance was used for comparison between multiple groups. The difference was statistically significant at *P* < 0.05.

## 3. Results

### 3.1. miR-22-3p Expression in CRC

**Figure 1:**
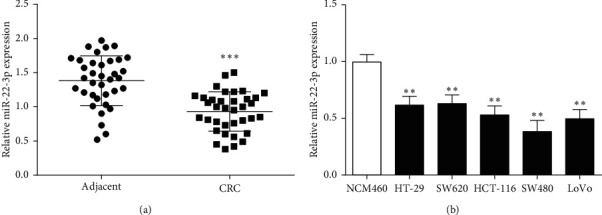
Expression of miR-22-3p was examined in CRC tissues and cells. (a) Expression of miR-22-3p was decreased in CRC tissues. (b) miR-22-3p expression in CRC cell lines. ^*∗∗*^*P* < 0.01 and ^*∗∗∗*^*P* < 0.001, compared with adjacent tissues or NCM460 group.

The expression level of miR-22-3p in CRC tissues was lower than that in adjacent tissue through qRT-PCR detection (Figure 1(a)). The detection of miR-22-3p in CRC cells showed that the expressions of five types of CRC cells (HT-29, SW620, HCT-116, SW480, and LoVo) were downregulated versus NCM460 cells (Figure 1(b)). Data suggested that miR-22-3p has poor expression in CRC. Herein, SW480 cells with the lowest expression in the above five cells were selected for further experimentation.

### 3.2. miR-22-3p Suppressed CRC Cell Proliferation and Invasion

**Figure 2:**
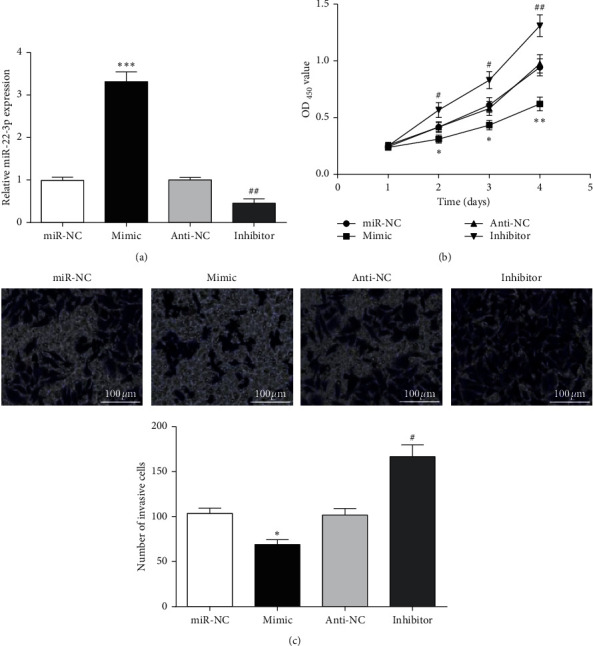
miR-22-3p regulated cell proliferation and invasion in CRC cells. (a) miR-22-3p expression in SW480 cells transfected with mimic or inhibitor. (b) Cell proliferation of SW480 cells after miR-22-3p knockdown or overexpression. (c) Cell invasion of SW480 cells after miR-22-3p knockdown or overexpression. ^*∗*^*P* < 0.05, and ^*∗∗*^*P* < 0.01, and ^*∗∗∗*^*P* < 0.001, compared with miR-NC group; ^#^*P* < 0.05 and ^##^*P* < 0.01compared with anti-NC group.

To investigate whether miR-22-3p dysregulation affects biological behavior of CRC cells, we transfected miR-22-3p mimic or inhibitor into SW480 cells to interfere in its expression. Compared to NC groups (miR-NC or anti-NC), miR-22-3p expression was increased in cells transfected with miR-22-3p mimics, while it was decreased by miR-22-3p inhibitor (Figure 2(a)). The CCK-8 assay was used to detect the cell proliferation of SW480 cells in each group. Results suggested that increasing miR-22-3p expression could inhibit the growth of SW480 cells, and miR-22-3p inhibition could significantly promote SW480 cell proliferation (Figure 2(b)). Similarly, Transwell experiments of each group showed that the number of cell invasions in mimic group was lower than that of miR-NC group, but it was higher in inhibitor group than in anti-NC group (Figure 2(c)). The results indicated that cell proliferation and invasion ability of SW480 was markedly enhanced after miR-22-3p expression was inhibited, while miR-22-3p overexpression reduced SW480 cell proliferation and invasion ability.

### 3.3. miR-22-3p Regulated RAP2B Expression

**Figure 3:**
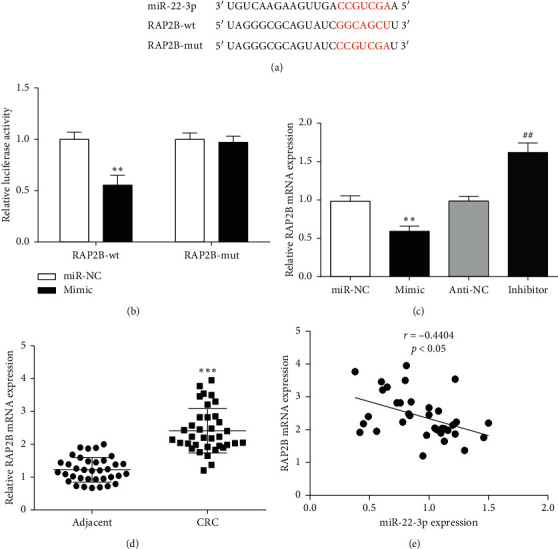
miR-22-3p directly targets RAP2B in CRC. (a) Predicted binding site between miR-22-3p and RAP2B. (b) The luciferase activity in SW480 cells. (c) RAP2B mRNA expression in SW480 cells after mimic or inhibitor transfection. (d) Expression of RAP2B in CRC tissue. (e) Correlation analysis between miR-22-3p and RAP2B in CRC tissues. ^*∗∗*^*P* < 0.01 and ^*∗∗∗*^*P* < 0.001, compared with miR-NC or adjacent group. ^##^*P* < 0.01, compared with anti-NC group.

Considering the effect of miR-22-3p on the behavior of CRC cells, we further analyzed its possible target genes and potential regulatory mechanisms. According to TargetScan online software analysis, we found that RAP2B may be one of the important target genes regulated by miR-22-3p. miR-22-3p can complementarily bind to the 3' UTR region of RAP2B (Figure 3(a)). The detection of luciferase activity revealed that miR-22-3p overexpression obviously reduced luciferase activity in the RAP2B-wt group but had no significant effect on the RAP2B-mut group (Figure 3(b)). This showed that miR-22-3p could bind to RAP2B.

To confirm the regulatory relationship of miR-22-3p and RAP2B, we detected the expression of RAP2B in SW480 cells overexpressing or silencing miR-22-3p. QRT-PCR results showed that the expression level of RAP2B was significantly elevated in miR-22-3p inhibition cells but was reduced by miR-22-3p overexpression (Figure 3(c)). This indicated that miR-22-3p may play a negative role in regulating RAP2B. Furthermore, RAP2B was also upregulated in CRC tissues (Figure 3(d)). More importantly, there was a negative correlation between RAP2B and miR-22-3p expression in CRC tissues (Figure 3(e)), suggesting that miR-22-3p may affect the occurrence and development of CRC by regulating RAP2B.

### 3.4. miR-22-3p Modulated Cell Proliferation and Invasion via RAP2B

**Figure 4:**
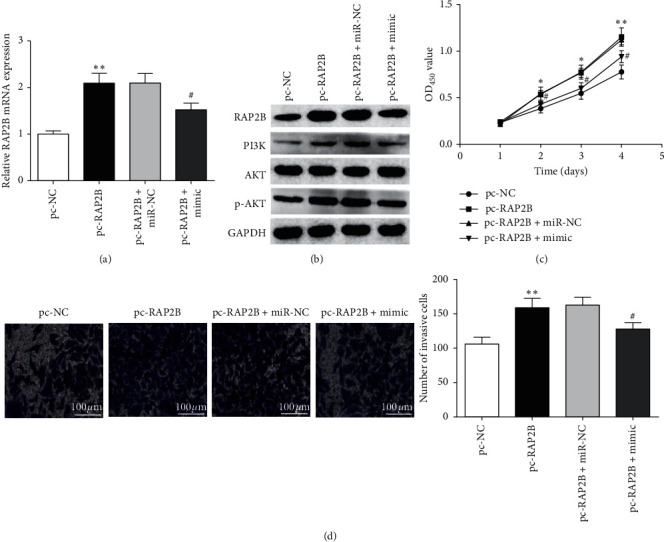
miR-22-3p could regulate SW480 cell proliferation and invasion through RAP2B. (a) RAP2B mRNA expression in SW480 cells after pc-PTEN and mimic cotransfection. (b) Protein levels of RAP2B, PI3K, AKT, and p-AKT in SW480 cells were determined by Western blot analysis. (c) Cell proliferation in SW480 cells cotransfected with miR-22-3p mimic and pc-RAP2B. (d) Cell invasion of SW480 cells cotransfected with miR-22-3p mimic and pc-RAP2B. ^*∗*^*P* < 0.05, and ^*∗∗*^*P* < 0.01 compared with pc-NC group; ^#^*P* < 0.05 compared with pc-RAP2B + miR-NC group.

Moreover, to verify whether miR-22-3p inhibited cell proliferation and invasion by RAP2B, SW480 cells were cotransfected with pc-RAP2B or pc-NC and miR-22-3p mimic or miR-NC. RAP2B expression was enhanced by pc-RAP2B, but enhancement was abolished by the cotransfection of pc-RAP2B and miR-22-3p inhibitor (Figures 4(a) and 4(b)). Additionally, PI3K and p-AKT protein levels were obviously increased in pc-RAP2B group, while this was reversed by miR-22-3p mimic cotransfection (Figure 4(b)); and there was no significant effect on AKT protein (Figure 4(b)). CCK-8 assay showed that miR-22-3p mimic abrogated the increasing cell proliferation caused by pc-RAP2B (Figure 4(c)). Additionally, miR-22-3p mimic also weakened the promotion of pc-RAP2B on cell invasion in SW480 cells (Figure 4(d)). To sum up, our data supported that miR-22-3p could contribute to CRC progression through RAP2B/PI3K/AKT pathway.

### 3.5. Identification of hBMSCs-Exosomes

**Figure 5:**
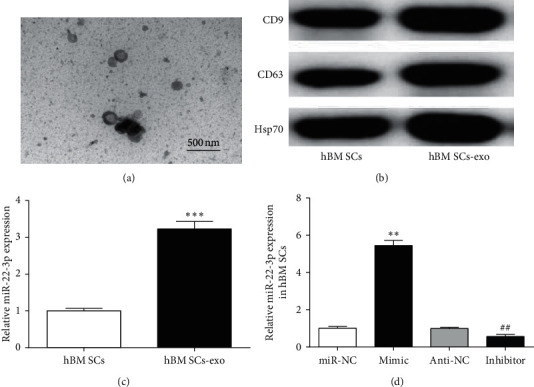
Identification of exosome sorted from hBMSCs. (a) The morphology of exosome was observed under transmission electron microscopy. (b) Surface markers (HSP70, CD63, and CD9) of hBMSCs-exosomes were detected by Western blot analysis. (c) miR-22-3p expression in hBMSCs and hBMSCs-exosomes. (d) miR-22-3p expression in hBMSCs treated with mimic or inhibitor. ^*∗∗*^*P* < 0.01 and ^*∗∗∗*^*P* < 0.001, compared with hBMSCs or miR-NC group; ^##^*P* < 0.01, compared with anti-NC group.

Exosomes were extracted from the hBMSCs culture medium by the ultracentrifugation. The extracted exosomes were identified through electron microscopy and Western blot. The results suggested that the observation of morphologies under the transmission electron microscope showed a typical exosome structure, that is, a cup-shaped vesicle-like structure with a complete double-layer lipid membrane coating between 40 and 100 nm in diameter (Figure 5(a)). Western blot detection showed that exosome surface markers CD63, Hsp70, and CD9 were expressed higher in hBMSCs-exo than in hBMSCs (Figure 5(b)). The morphology and molecular level confirmed that the extracted vesicles were exosomes.

Subsequently, we detected miR-22-3p expression in hBMSCs and hBMSCs-exosomes. We found that miR-22-3p expression was enhanced in hBMSCs-exosomes (Figure 5(c)). To confirm whether hBMSCs-derived exosomes are rich in miR-22-3p, miR-22-3p expression was also measured in transfected hBMSCs. miR-22-3p was obviously upregulated in hBMSCs treated with miR-22-3p mimic, while it was downregulated in miR-22-3p inhibitor treatment hBMSCs (Figure 5(d)). In conclusion, exosomes derived from hBMSCs which were transfected with miR-22-3p mimics could effectively overexpress miR-22-3p, providing a good basis for subsequent experiment.

### 3.6. miR-22-3p from hBMSCs-Exosomes Inhibited Cell Proliferation and Invasion

**Figure 6:**
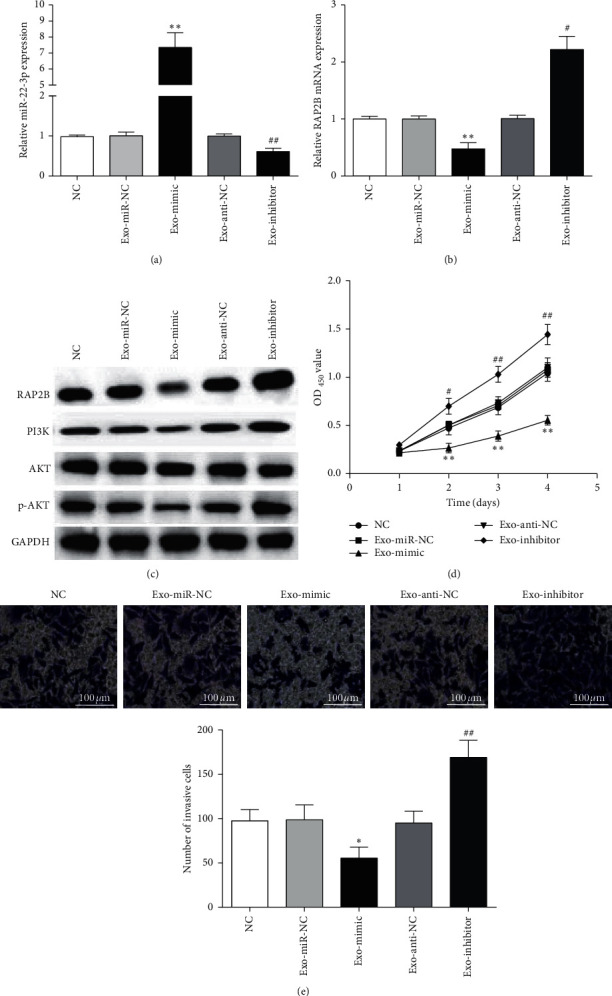
Exosomal miR-22-3p regulates SW480 cells proliferation and invasion via RAP2B. (a, b) miR-22-3p and RAP2B expression in SW480 cells. (c) Protein levels of RAP2B, PI3K, AKT, and p-AKT in SW480 cells. (d, e) SW480 cells proliferation and invasion were measured by CCK-8 and Transwell assays. ^*∗*^*P* < 0.05 and ^*∗∗*^*P* < 0.01, compared with exo-miR-NC group; ^#^*P* < 0.05 and ^##^*P* < 0.01, compared with exo-anti-NC group.

To verify whether miR-22-3p from hBMSCs-exosomes regulate CRC cell biological function, CCK-8 and Transwell assays were conducted. Expression of miR-22-3p was markedly enhanced and RAP2B expression was notably reduced in SW480 cells treated with exo-mimic (Figures 6(a) and 6(b)). However, the opposite trend of miR-22-3p and RAP2B expression was observed in SW480 cells treated with exo-inhibitor (Figures 6(a) and 6(b)). Moreover, exo-mimic reduced RAP2B, PI3K, and p-AKT protein levels, all of which were opposite in exo-inhibitor group (Figure 6(c)). Besides, the cell proliferation and invasion of SW480 were observably decreased after exo-mimic treatment, while exo-inhibitor could notably promote SW480 cell proliferation and invasion (Figures 6(d) and 6(e)), suggesting that miR-22-3p derived from hBMSCs exosome repressed SW480 cell function.

## 4. Discussion

CRC is one of the most common malignant tumors in China, and its incidence and mortality have increased year by year [19]. CRC has high proliferative and antiapoptotic properties, and this is one of the main reasons for its poor prognosis [20, 21]. Although the progression of CRC is a multistage and multifactor participation process, gene mutations and epigenetic changes are still the decisive factors for starting and promoting the CRC progression [22]. Therefore, it has great significance to find effective diagnostic molecular biomarkers and therapeutic targets for CRC.

In recent years, more and more evidence indicated that MSCs play an important role in the development and metastasis of certain tumors and that paracrine factors secreted by them may participate in these reactions [23]. Exosomes, as paracrine factors, carry a large number of bioactive molecules and transfer their contents to adjacent tumor cells, thereby inducing phenotypic modification of the recipient cells [24, 25]. The research of the interaction between miRNAs from hBMSCs-exosomes and tumor has become a hot topic. Some functional RNAs can be transferred into tumor cells by exosomes and play roles in some physiological and pathological processes, including miRNAs [26]. Our current study explored the role of hBMSCs-exosome-miR-22-3p in CRC.

Currently, there are few studies on miR-22-3p in CRC, let alone its mechanism. Zhang et al. reported that miR-22 expression was reduced in CRC tissues, which was associated with liver metastasis and poor overall survival [27]. Sha et al. also found that miR-22-3p was downregulated in CRC and that LINC00858 could directly target it to regulate its target gene YWHAZ, thus promoting CRC progression [28]. Our results showed that the expression of miR-22-3p in CRC was decreased, which is in consistency with previous studies. In order to explore the mechanisms of miR-22-3p in CRC, RAP2B was predicted and confirmed as its target genes. Besides, RAP2B was upregulated and negatively correlated with miR-22-3p expression in CRC tissues. miR-22-3p overexpression inhibited the expression of RAP2B, suggesting that miR-22-3p targeted and negatively regulated RAP2B.

RAP2B located on 3q25.2 is an oncogene that is highly expressed in a variety of tumors and plays an important role in promoting biological processes (such as tumor cell proliferation, metastasis, etc.) [29, 30]. In this study, RAP2B overexpression notably promoted CRC cell proliferation and invasion, and the facilitation was offset by miR-22-3p overexpression. Besides, PI3K and p-AKT protein levels were obviously increased by RAP2B overexpression, while this was reversed by miR-22-3p overexpression. Thus, we suggested that miR-22-3p overexpression repressed cell proliferation and invasion of CRC cells through RAP2B to inactive PI3K/AKT pathways.

Furthermore, we found that the exosomes derived from hBMSCs effectively expressed specific markers CD63, Hsp70, and CD9 and contained abundant miR-22-3p after miR-22-3p mimic treatment. It was found that exosomes from hBMSCs treated by miR-22-3p mimic enhanced miR-22-3p and reduced RAP2B expression in CRC cells, while the opposite trend was observed for exosomes from hBMSCs treated with miR-22-3p inhibitor. Moreover, the cell proliferation and invasion ability of CRC cells were observably repressed by miR-22-3p derived from hBMSCs exosome. Besides, p-AKT protein level was also inhibited by hBMSCs-exosome-miR-22-3p. All of above results were similar to those in the transfection miR-22-3p mimic or inhibitor. The findings indicated that miR-22-3p carried by hBMSCs-exosome played a suppressor role of CRC via RAP2B/PI3K/AKT pathway.

Although our study verified that exosome-miR-22-3p exerted a suppressor role in CRC, some shortcomings still exist. If adequate clinical samples, in vivo animal experiments, and other potential target genes or signaling pathways (key protein expression level in pathway, especially in PI3K/AKT pathway) are added, the result could be more credible. Thus, there is still a need to conduct further clinical verification and experiments.

## 5. Conclusions

Collectively, we demonstrated that exosome-miR-22-3p derived from hBMSCs suppressed CRC cells proliferation and invasion via suppressing RAP2B expression to inhibit PI3K/AKT pathway, proposing that miR-22-3p might become a potential marker for early diagnosis and treatment of CRC patients.

## Figures and Tables

**Table 1:** Primer sequences for real-time fluorescence quantification PCR.

Gene name	Primer sequences (5′-3′)
*GAPDH*	F ACGCTGCATGTGTCCTTAG
	R GAGCCTCTTATAGCTGTTTG

*U6*	F CTCGCTTCGGCAGCACA
	R AACGCTTCACGAATTTGCGT

*miR-22-3p*	F AAGCTGCCAGTTGAAGAACTGTA
	R GCTGTCAACGATACGCTACGTAAC

*RAP2B*	F CTGCCCCTTCATGGAGACA
	R TGCGAATAGCTCATCCACTGA

## Data Availability

The datasets used and/or analyzed during the present study are available from the corresponding author on reasonable request.
